# Role of Cannabinoids in Obesity

**DOI:** 10.3390/ijms19092690

**Published:** 2018-09-10

**Authors:** Francesca Rossi, Francesca Punzo, Giuseppina Rosaria Umano, Maura Argenziano, Emanuele Miraglia Del Giudice

**Affiliations:** Department of Woman, Child, General and Special Surgery, University of Campania “Luigi Vanvitelli”, 80138 Napoli, Italy; francesca.punzo19@gmail.com (F.P.); giusi.umano@gmail.com (G.R.U.); maurargenziano@gmail.com (M.A.); emanuele.miragliadelgiudice@unicampania.it (E.M.D.G.)

**Keywords:** CB1, CB2, obesity, WAT, BAT, browning, beige adipocytes, adipocytes, cannabinoids

## Abstract

Obesity is an increasing health problem worldwide. Its related comorbidities imply a high cost for the National Health System and diminish a patient’s life quality. Adipose tissue is composed of three types of cells. White adipocytes are involved in fat storage and secretion of hormones. Brown adipocytes are involved in thermogenesis and caloric expenditure. Beige adipocytes are transitional adipocytes that in response to various stimuli can turn from white to brown and could be protective against the obesity, enhancing energy expenditure. The conversion of white in beige adipose tissue is a potential new therapeutic target for obesity. Cannabinoid receptors (CB) regulate thermogenesis, food intake and inflammation. CB1 ablation or inhibition helps reducing body weight and food intake. Stimulation of CB2 limits inflammation and promotes anti-obesity effects by reducing food intake and weight gain. Its genetic ablation results in adiposity development. CB receptors are also responsible for transforming white adipose tissue towards beige or brown adipocytes, therefore their modulation can be considered potential anti-obesity target. CB1 principal localization in central nervous system represents an important limit. Stimulation of CB2, principally localized on peripheral cells instead, should facilitate the anti-obesity effects without exerting remarkable psychotropic activity.

## 1. Obesity

Obesity is a public health problem. Worldwide, nearly 1.9 billion adults are overweight and 600 million are obese [1]. It has been reported that about 50 million girls and 74 million boys were obese in 2016 and its prevalence has dramatically risen in recent decades. Strong evidence report that obese children are more likely to become obese adults. Recently, Ward and coworkers reported that about the 74% of 2-year-old obese children and 88% of 19-year-old obese youth are still obese at 35 years of age [2]. To date, lifestyle intervention constitutes the first line of obesity treatment. However, it is limited by low efficacy and high drop-out rates. Currently, only one medication, namely Orlistat, has been approved by the Food and Drugs Administration (FDA) for weight loss in children and adolescents. In adults, the FDA have approved six different drugs for obesity treatment: Orlistat [3,4,5,6,7], Phentermine/Topiramate [7,8,9,10,11,12], Naltrexone/Bupropion [13,14,15,16,17], Lorcaserin [7,18,19,20] and Liraglutide [7,21,22,23,24]. These medications share common pharmacodynamical mechanisms, except for Orlistat which reversibly inhibits the pancreatic and gastrointestinal lipases, increasing dietary fat excretion.

The endocannabinoid system (ECS) is known to regulate several metabolic processes, such as food intake and the energy expenditure. It encompasses Cannabinoid receptors type 1 and 2 (CB1 and CB2), their endogenous ligands and enzymes for their synthesis and inactivation. CB1 is the most abundant G-protein-coupled receptor in the central nervous system, especially in the hippocampus, cortex, cerebellum, and basal ganglia. CB2 is predominately expressed in the spleen, thymus and circulating immune cells, but also in skeletal, cardiovascular, and renal systems. ECS receptors are also expressed in bone tissue, where they stimulate bone formation and remodeling, and in adipose tissues, where they directly influence lipid metabolism in vitro [25,26].

Adipose tissue depots are commonly distinguished in white and brown adipose tissue according to their appearance. Brown adipose tissue (BAT) is characterized by small lipid droplets and high density of mitochondria which leads to the brown appearance. White adipose tissue (WAT) cells show a unilocular lipid droplet. The morphologic differences reflect different functions. BAT is involved in thermogenesis and caloric expenditure during resting and exercise by the mitochondrial uncoupling protein-1 (UCP1), which uncouples oxidative phosphorylation from ATP production [27]. WAT is involved in fat storage and endocrine secretion of hormones. In response to various stimuli, UCP1-expressing multilocular adipocytes develop in WAT. These are beige or brite adipocytes and their induction or recruitment, together with the activation of BAT, could be protective against obesity [28] enhancing body energy expenditure. Several activators have been associated with WAT browning, namely cold, exercise, thyroid hormones, catecholamines, capsaicin etc. The conversion of WAT in beige adipose tissue is a potential new therapeutic target for obesity. It might increase the resting energy expenditure improving the energy balance. The ECS is known to be involved in controlling energy metabolism, thermogenesis, and inflammation [25,29]. Through a revision of the literature on this system in obesity, we evaluate its role and potential as therapeutic target in this pathology.

## 2. The “Browning” Process and Cannabinoid Receptors Involvement

BAT activity has the potential to significantly influence body weight, glucose and lipid metabolism [30]. It is involved in dissipating energy as heat, due to the UCP1 on the inner mitochondria membrane. UCP1 promotes the free flux of proton across the inner mitochondria membrane skipping the production of ATP. Therefore, brown adipocytes might be responsible for large heat production, significantly higher than in other organs [31].

Studies have been conducted to investigate the line tracing and to clarify whether white and brown adipocytes share a common precursor. Mesenchymal stem cells give rise to adipose tissue, muscle, and bone. White adipocytes are characterized by PPAR-γ (peroxisome proliferator-activated receptor-γ) expression and activation. PPAR-γ is a transcriptional factor that promotes the expression of genes involved in adipogenesis and triglycerides accumulation. Other factors involved in WAT differentiation are C/EBP (CCAAT/enhancer binding protein) family (α, β, δ) Krox20, KLFs, and EBFs. Conversely, brown adipocytes are characterized by Myf5 (myogenic factor 5) expression. Experimental studies have proven that brown adipocytes share a common precursor with myocytes. The activation of the transcriptional factor PRDM16 (PR domain containing 16) promotes the *Myf5* expression and the differentiation towards brown adipocytes, whereas the reduction of PRDM16 enables the myogenic program. In addition, PRDM16 pathway suppresses WAT genes and activates BAT genes. Other important transcriptional factors for the thermogenic activity are PPAR-α and Pgc1-α [32].

The EC system is deeply involved in controlling energy metabolism; in particular the CB1 receptor is involved in controlling lipid and glucose metabolism. Even though CB1 is principally expressed in the nervous system and its expression levels are very low in peripheral cells, it increases in obesity. Genetic ablation of CB1 results in a reduction of body weight as well as its selective blockade reduces food intake and body weight too. Also, CB2 plays a role in feeding; in particular its agonists can reduce food intake and its genetic ablation results in adiposity development. In the literature there is considerable evidence about the negative impact that CB1 has on thermogenesis. It was observed that CB1-lacking mice have less fat and are more protected against obesity than the correspondent wild-type mice [33]. These data suggest that the blockade of CB1 receptor could induce the trans-differentiation of white adipocytes towards a thermogenic brown cell phenotype, even though it is not always observed a statistically significant increase in lipid accumulation after incubation with Rimonabant (inverse agonist at CB1) [34,35].

**Figure 1:**
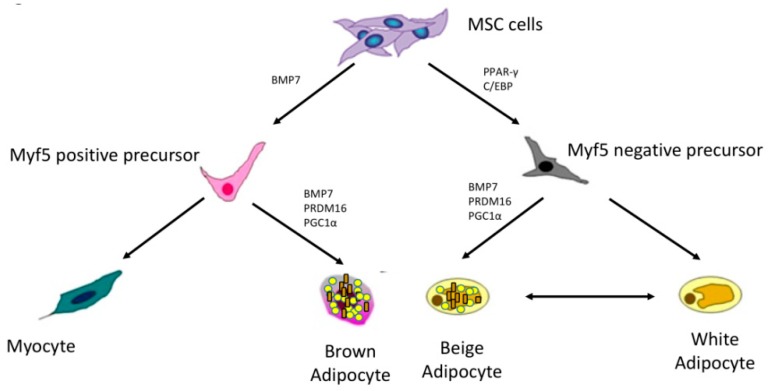
Brown, white, and adipocytes lineages. (Mesenchymal stem cells (MSC) give rise to myogenic factor 5 (Myf5) positive and negative precursors. Myf5 positive precursors generate brown adipocytes under the effect of bone morphogenetic protein 7 (BMP7), PR domain containing 16 (PRDM16) and peroxisome proliferator-activated receptor gamma coactivator 1-α (PGC1α). Myf5 negative precursors, under the effect of peroxisome proliferator-activated receptor (PPAR-γ) and CCAAT/enhancer binding protein (C/EBP), generate white and beige adipocytes).

Nevertheless, it has been reported that WAT might develop brown fat-like characteristics, a process referred as “browning”. The first evidence came from mouse models. In 1984, Young et al. reported that cold exposure in mice led to an increase of brown-like fat cells and UCP1 expression in parametrial fat depots that are usually constituted of white adipose cells [36]. Later, other environmental stimuli have been associated with brown fat-like activity of WAT in mice acting via adrenergic stimulation (exercise, stress, thyroid hormones, irisin, and adrenoreceptor agonists) [37]. This intermediate adipocytes population is referred as “beige” or “brite” (brown in white) adipose tissue. Beige adipocytes express UCP1 and are capable of dissipating energy as heat as well as BAT in response to environmental cues [38]. In addition, beige cells express various transcriptional factors that are crucial for brown functioning, such as PRDM16 and PGC1α (peroxisome proliferator-activated receptor gamma coactivator 1-α). Although beige and brown adipocytes share similar characteristics, evidence suggests that they originate from different precursors [39,40]. It has been hypothesized that a subset of WAT precursor (Myf5 negative) might generate inducible beige adipocytes. Several mediators such as BMP7 (bone morphogenetic protein 7), PRDM16, PGC1α have been associated with the development of inducible beige adipocytes and trans-differentiation of white cells to beige cells [41] (Figure 1).

Both brown and beige adipocytes have the potential to positively influence energy balance and metabolic profile due to their thermogenic activity. In humans, population studies have reported that BAT might be activated by prolonged cold exposure (19 °C). BAT activity is enhanced in winter compared to summer [42]. BMI (body mass index), visceral and total body fat are inversely associated with BAT activation, with obese subjects having less BAT compared to lean. Moreover, metabolically active BAT is inversely associated with plasma glucose and lipids levels [43]. In 2014, Chondronikola and coworkers reported that adult men with active BAT showed a significant increase of resting energy expenditure compared to BAT negative men. BAT activation was supported by plasma glucose and free fatty acids oxidation. Moreover, subjects with active BAT showed higher glucose disposal and insulin sensitivity compared to the group with no BAT [44]. These findings emphasize the potential role of browning in addressing the global epidemiology of obesity and its related comorbidities. The recent evidence of beige fat in humans supports this hypothesis.

In 2016 Lucia M. Krott et al. [45] investigated the role of EC system in obese mice under conditions that are known to cause BAT activation and WAT browning (i.e., cold exposure), observing the upregulation of endocannabinoids (ECs) and of enzyme biosynthesis in WAT together with the CB1 inhibiting action on thermogenesis and lipid droplet formation [46].

Recently Minna Lahesmaa et al. (2018), reported a positive correlation between CB1 receptor density and glucose uptake in human BAT under cold exposure. The measurements have been performed by Positrone-Emission Tomography (PET) imaging methods with high sensitivity [47]. This and much other evidence in the literature suggests the possibility of using CB1 receptor as a therapeutic target to treat obese subjects, even though its principal localization, in the central nervous system, represents an important limit. The main side effects caused by drugs such as Rimonabant were hypothermia, analgesia, hypo-locomotion, and catalepsy. Hence the necessity to individuate novel therapeutic approaches. Hsiao et al. [48] tested the efficacy of a second-generation CB1 antagonist (BPR0912) that presents an almost exclusively peripheral distribution. It has been demonstrated that these kind of drugs enhance the formation of UCP1-expressing cells in WAT, therefore are able to induce the shift of WAT cells towards the beige phenotype (browning process), by only blocking the peripheral CB1 receptor [29]. These groups of compounds act centrally by blocking CB1 receptor and thus reducing food intake stimuli, but also peripherally by enhancing thermogenesis with energy expenditure, so they could be a potential suitable target.

**Figure 2:**
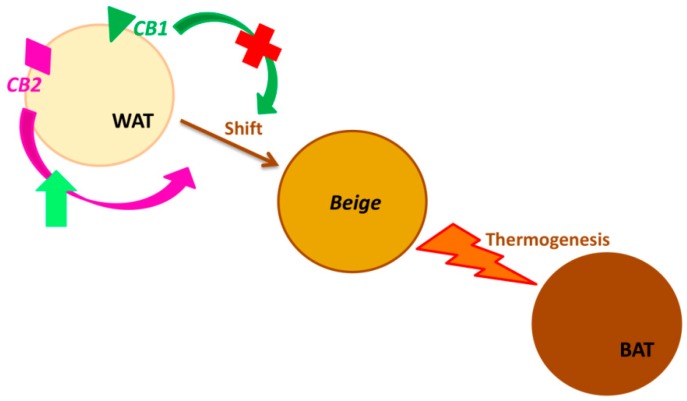
Cannabinoid receptors (CB1 and CB2) in Browning. (Browning is the process by which the white adipose tissue’s physiology and morphology switch from white (WAT) towards brown phenotype (BAT), through the intermediate beige phenotype. CB2 receptor stimulation enhances this transformation, triggering the thermogenesis. Analogously, the blockade of CB1 receptor induces the same adipocyte’s phenotype change).

The effects of CB2 receptor on obesity are poorly characterized, because only recently was its localization observed in other sites than immune cells: liver, adipose tissue, pancreatic islet cells. These findings highlighted the CB2 involvement in energy homeostasis [49]. Rossi et al. [50] demonstrated that the adipocytes from obese subjects express significant low levels of UCP1 and that these levels increase in a statistically significant manner after the stimulation of CB2 with its agonist JWH-133, thus increasing the heat generation with consequent energy expenditure (Figure 2). In their study, Verty at al. [49] reported that while the inhibition of CB2 receptor signaling by injection of AM630 (antagonist at CB2) produces a significant increase in food intake in non-obese rodents, chronic stimulation of CB2 with JWH-015 (agonist at CB2) attenuates body weight gain. Moreover, they observed that this agonist has no effect on UCP1 expression levels but induces the increase of some lipolysis markers (i.e., ATGL) in WAT.

D.A. de Luis et al. [51] demonstrated some effects of polymorphism rs3123554 of CB2 receptor on adiposity, observing that the carriers of this genic variant loose less body weight under hypo-caloric diet. Also, this finding could confirm the role of CB2 stimulation in increasing thermogenesis and in enhancing browning process. All these findings are interesting, above all, because they suggest the possibility to further investigate the possibly therapeutic effects of CB2 stimulation to revert obese-related conditions, by-passing the use of drugs acting on CB1 and expressing psychotropic side effects.

## 3. CB1 and CB2 in Food Intake

Adipose tissue regulates many physiological processes, being an important endocrine organ. Proteins such as leptin, lipoprotein lipase and adiponectin are physiologically produced by the adipose tissue. The leptin has an important role in food intake, bodyweight control and metabolism. It modulates neuronal signaling pathways in the hypothalamus acting as anorexigenic mediator [52]. It has been demonstrated that hypothalamic concentrations of cannabinoids are inversely correlated with plasma concentrations of leptin, so they show orexigenic functions [26]. Antagonists at CB1 have been demonstrated to restore hypothalamic leptin sensitivity reducing obesity in diet-induced obese (DIO) mice [33]. Conversely, leptin reduces endocannabinoid synthesis by lowering intracellular calcium levels and the release of CB mediated by glucocorticoids [53,54]. There is an intricate interaction between cannabinoids and glucocorticoid systems since ECs signaling mediates many of the neurobiological and physiological effects of glucocorticoids (GCs). Conversely, GCs mobilize ECs to execute their function as mediators of homeostasis. The interaction between GCs and ECs is observed in several studies, for example in 2010 Wamsteeker et al. [55] demonstrated that GCs downregulate CB1 receptor expression [56] through genomic signaling, while through non-genomic signaling GCs enhance the ECs signaling. In particular, it has been demonstrated that GCs influence the ECS by mobilization of anandamide (AEA) and 2-arachidonoylglycerol (2-AG) [57,58]. AEA mediates effects in both central and peripheral nervous system, acting as ligand of CB1 in the central nervous system and of CB2 in peripheral cells. Leptin infusions in rats significantly decreased AEA levels in WAT [59]. 2-AG is the primary endogenous ligand for the CB2 receptor and an endogenous agonist of the CB1 receptor. 2-AG has been demonstrated to downregulate leptin expression and that CB1 and CB2 antagonists can invert this process, suggesting that CB receptors regulate the 2-AG related leptin expression. However, there is strong evidence that glucocorticoids modify feeding through neural pathways that involve the EC system, even though the principal significant interaction of GCs is with insulin [60]. It has been demonstrated that appropriate concentrations of general corticosteroids have stimulatory effects on caloric intake and also on food preference, in the presence of insulin [61].

Another interesting aspect is that a maternal high-fat (HF) diet could influence the ECS composition in BAT of mice offspring at birth, contributing to develop hyperphagia, food preference and higher adiposity later in life. In particular, it has been demonstrated a sex prevalence of these effects with a decrease in leptinemia together with an increase of CB1 and orexin-A in male pups, while maternal HF diet itself increases hypothalamic CB2 in female pups [62]. In the literature, impaired leptin signaling is associated with over activation of the central EC system, contributing to obesity development [63,64]. Low levels of leptin are correlated with a higher risk of obesity in humans too [65]. CB1 regulates cell proliferation, differentiation, and survival of neuron progenitors in the central nervous system. According to these findings, alterations in leptin and endocannabinoid signaling at birth could cause an altered hypothalamic development in HF offspring. The antagonism at CB1 in adipocytes also promotes the *trans*-differentiation of white adipocytes to brown, enhancing thermogenesis and activating the glucose use in DIO mice [66], even though there is considerable evidence that the activation of ECS in human adipocytes promotes the glucose uptake independently of insulin. Therefore, the focal point is the possibility to use antagonists at CB1 in anti-obesity treatments. Rimonabant, antagonist at CB1, has long been used as an anti-obesity drug able to induce weight loss, improve blood lipid parameters and increase the adiponectin level in obese patients [67], but it had severe side effects including depression, anxiety, nausea and dizziness probably due to the blockade of central CB1. Because of these complications Rimonabant has been withdrawn from the market. It is very important to overcome the possible psychological side effects and it could be useful to identify or to develop compounds with limited brain penetration but still maintaining the potential therapeutic efficacy. Less information is given about the CB2 receptor role in the central and peripheral control of energy metabolism. This receptor is prevalently expressed on immune cells and it is involved in inflammatory responses. CB2 receptor stimulation promotes anti-obesity effects by reducing food intake and weight gain without an adverse impact on mood and inhibiting activated macrophages and T cells. Recent evidence indicates that CB2 receptors, even if at much lower levels compared with CB1, are also expressed in the brain and involved in neuropsychiatric functions. Interestingly, it has been shown that only chronic activation of CB2 increases excitatory synaptic transmission, whereas its short-term activation has little effect on synaptic activity [60,68]. A therapeutic use of CB2 as anti-obesity target might presume a related chronic neuronal activation, that, in turn, increasing excitatory synaptic transmission, should facilitate the peripheral anti-obesity effects without exerting remarkable psychotropic activity. Nevertheless, selective CB2 agonists that cannot cross-over the hemato-encephalic barrier could be designed.

## 4. CB1 and CB2 in Obesity-Related Inflammation

Adipocyte hypertrophy and hyperplasia, impaired extracellular matrix remodeling and altered secretion of adipokines are presented together with increased inflammation in obesity. The association between obesity and chronic inflammation is suggested by several observations, such as the production of TNF-α and IL-6 by adipocytes in obese mice and humans as well as the presence of immune cells (i.e., macrophages) in adipose tissue. The secretion of pro-inflammatory cytokines from adipose tissue is associated with the risk of adverse outcomes in obesity (type 2 diabetes, cardiovascular diseases, nonalcoholic fatty liver disease and cancer) [69]. It was observed that IL-6 and TNF-α inhibit the expression of adiponectin [70], which is an adipokine, inversely correlated with the obesity [71], its increase plays an important anti-inflammatory role.

In the literature the role of EC system in inflammation has been deeply investigated and there is much evidence suggesting that an altered activation of the EC system causes an impairing in lipid metabolism, facilitating the progression of inflammation [72]. In particular, the anti-inflammatory role of CB2 receptor is well known. In 2016 Rossi et al. [50] demonstrated that a common missense CB2 variant (Q63R), which is less functional, is associated with a high *z*-score body mass index in a population of obese Italian children. Indeed, reverse agonists at CB2 lead to an increase inflammatory adipokine release together with fat storage, while the stimulation of CB2 reverses all the obesity-related effects. These observations suggest the possibility of using CB2 as a novel anti-obesity pharmacological target.

Lipid endocannabinoid signaling, through CB2 receptor, is a part of a protective system: inflammation causes an increase of EC system elements, which in turn regulate immune cells. Changes in EC receptors expression levels have been reported in almost all diseases affecting humans [73], which highlight the crucial role of the EC system in regulating several biological pathway. In particular, the activation of CB2 receptor mediates immunosuppressive responses, limits inflammation as well as tissue-associated injury in large number of pathological conditions, even though it was observed that in some diseases the activation of CB2 itself could enhance tissue damage. In their review article Pachera and Mechoulamb (2010) [73] defined CB2 as a “cannabinoid receptor with an identity crisis”, due to the discordant data in the literature about its role. Agudo et al. [74] demonstrated that adipose tissue hypertrophy was not associated with inflammation in mice and moreover 2-month-old CB2 –/– mice under HF diet showed a normal insulin sensitivity and no body weight gain. These results indicated that the lack of CB2 has protective effects, which implies that it could be a potential target to contrast obesity and insulin resistance. Also, Deveaux et al. [75] observed that the induction of CB2 receptor in the adipose tissue is correlated with an increase of fat storage and inflammation. In these cases, data suggest that CB2 receptor antagonists could represent a novel therapeutic approach for obesity-associated metabolic disorders. However, in the literature the data suggesting anti-inflammatory properties of CB2 are more representative, Xu et al. also reported anti-inflammatory properties of a common CB2 receptor agonist, JWH-133 in mice, attributing them to the inhibition of the auto-reactive T cells with the consequent prevention of leukocyte trafficking into the inflamed tissue [76].

## 5. Conclusions

Obesity-related co-morbidity implies a high cost for society and reduces a patient’s lifestyle quality [1,77,78,79,80,81]. Since an obese child will likely be an obese adult, planning childhood intervention on obesity would be the most advisable solution. Diet intervention and correct lifestyle are surely the best solution, but dietary regiments are negatively affected by a strong cultural influence, especially in certain areas of the world, therefore it is sometimes not an efficient approach. So far only one treatment has been approved by the FDA for weight loss in children and adolescents (Orlistat), and not without important side effects, such as gastrointestinal discomfort, flatulence, oily stools, fecal urgency, stool incontinence, liposoluble vitamin deficiency, pancreatitis, nephrotoxicity and hepatotoxicity [3]. We invite researchers to investigate new pharmaceutical targets suitable for children and young adults. The recent findings in the field of browning in humans have pointed out the potential role of this phenomenon in obesity treatment. In addition, BAT activity has been associated with an improving of metabolic profile in adults [30], although no large clinical data are available at the time. In light of what has been discussed so far, CB2, thanks to its capacity of enhancing browning and transforming chemical energy into thermic energy, could be a possible solution, rather than targeting CB1 which, as discussed previously, causes psychotropic effects [50]. CB2, instead, could show a higher safety profile with negligible side effects.
